# Isolation, screening, preliminary optimisation and characterisation of thermostable xylanase production under submerged fermentation by fungi in Durban, South Africa

**DOI:** 10.1080/21501203.2022.2079745

**Published:** 2022-06-20

**Authors:** Priyashini Dhaver, Brett Pletschke, Bruce Sithole, Roshini Govinden

**Affiliations:** a Discipline of Microbiology, School of Life Sciences, Westville Campus, University of KwaZulu-Natal, Durban, South Africa; b Enzyme Science Programme (ESP), Department of Biochemistry and Microbiology, Rhodes University, Eastern Cape, South Africa; c Biorefinery Industry Development Facility, Council for Scientific and Industrial Research, Durban, South Africa; d Discipline of Chemical Engineering, University of KwaZulu-Natal, Durban, South Africa

**Keywords:** Fungi, xylanase, screening, isolation, xylan plate assay

## Abstract

Fungi are renowned for their ability to produce extracellular enzymes into their surrounding environment. Xylanases are hydrolytic enzymes capable of xylan degradation. The objectives of this study were to isolate, screen for potential xylanolytic fungi from soil and tree bark samples from three locations in South Africa and to determine their growth conditions for maximum xylanase production. Forty-six isolates were obtained based on clearing zone formation on xylan-enriched agar plates using Congo red indicator. Xylanase activity was quantified during submerged fermentation. Isolate MS5, identified as *Trichoderma harzianum* with the highest enzyme activity (38.17 U/ml) was selected for further studies based on thermophilic properties (70°C) and pH (5.0). The culture conditions; incubation period (5 days), agitation speed (160 rpm) wheat bran (1%) and ammonium sulphate (1.2%) were optimised further. Biochemical characterisation of the crude enzyme revealed two pH and temperature optima (pH 6.0 at 60°C and 70°C, pH 8.0 at 55°C and 75°C). The enzyme retained >70% activity after 4 h at pH 6.0 at 70°C. SDS-PAGE revealed multiple protein bands with a prominent band at 70 kDa. Substrate Native PAGE revealed multiple isoforms between 55 and 130 kDa. This enzyme will be beneficial for applications in the animal feed and biofuels industries.

## 1. Introduction

Lignocellulosic materials are widespread in nature and xylan is a polysaccharide found in the hemicellulose fraction of lignocellulose, a major component of the plant cell wall. Xylan is a significant resource of renewable biomass, suitable as a substrate for the production of many commodities such as biofuels, affordable energy sources for fermentation and improved animal feeds. However, xylan must be converted to xylose and xylo-oligosaccharides for most bioconversion processes. The conversion of xylan can be performed by acid hydrolysis or by xylanolytic enzymes (xylanases) that deconstruct plant structural material thus breaking down hemicellulose (Bhardwaj et al. 2019).

Xylanase production by fungi, bacteria, yeast, marine algae, etc. has been studied and reported by several authors, but the main sources of industrial enzymes are fungi and bacteria (Wong et al. 1988; Prade 1996; Mandla 2015; Lee et al. 2018) through intracellular or extracellular secretions by the microorganisms (Lee et al. 2018). Depending on the source, xylanases have different characteristics, which make them useful for several applications. Microbial enzymes are preferred in industrial applications due to their ability to be produced in large volumes over a short period (Adesina and Onilude 2013; Motta et al. 2013). Fungi are highly diverse in nature and have been recognised as an unrivalled target for enzyme screening (Nair and Shashidhar 2008). Filamentous fungi are producers of xylanases and other xylan degrading enzymes, with the noteworthy characteristics of secreting enzymes into the surrounding medium, thus avoiding the need for cell lysis, and with much higher activities compared to yeasts and bacteria (Nair and Shashidhar 2008). Thus, fungal enzymes are very attractive for various industrial processes (Prasad Uday et al. 2017). On an industrial scale, xylanases are produced mainly by *Aspergillus and Trichoderma* spp. in solid-state fermentation (SSF). *T. harzianum* is present in all soil types and are the most prevalent culturable fungi (Chen and Zhuang 2017).

The application of xylanases in industrial processes has had many limitations for its commercial feasibility due to several factors (Walia et al. 2013). These include the inaccessibility of substrate to the xylanases caused by physical limitations, the incomplete hydrolysis of xylan due to its diverse branched nature, the narrow pH optimum range, thermal instability of the enzymes, end-product inhibition, and cost of enzyme production (Walia et al. 2013). Fungal xylanases are effective in a pH range of 4.0–6.0 and temperatures below 50°C, thus their use in industrial applications is restricted. Previous studies also showed that mesophilic organisms are not ideal for xylanase production as these enzymes generally become denatured at temperatures above 55°C (Robledo et al. 2016). As a consequence, the efficiency of hydrolysis decreases during the catalytic application, requiring supplementation during the process or higher enzyme yields to overcome this problem, thus increasing the process costs. Therefore, the use of thermostable enzymes is essential for hydrolysis at high temperatures.

Xylanases have been used in the feed industry to reduce the viscosity of food and improve nutrient absorption in the digestive tracts of animals (Bedford 2018). The enzymes could be applied when the feeds are being processed, before the pellet process (70–95°C, pH 4–6), indicating the requirement for thermostable enzymes that are also active in acidic conditions (Collins et al. 2005; Pariza and Cook 2010). Numerous studies have been conducted to isolate thermophilic enzymes with superior enzyme stability from fungi (Haki and Rakshit 2003; Robledo et al. 2016). Thermophilic xylanases are characterised by temperature optima between 50°C and 80°C and are stable in this range (Haki and Rakshit 2003). Chadha et al. (2019) published a review on thermophilic fungal and bacterial xylanases that details that these enzymes have a multiplicity of isoforms (in excess of 15 for *Myceliophothora sepedonium*) in some instances that was revealed by transcriptomic and proteomic studies. However, due to the yield of enzymes required for large-scale applications, the search for microorganisms able to produce thermostable xylanases with high yields and characteristics desired for industrial applications is still ongoing. Considering the industrial importance of xylanase, the aim of this study was to isolate, screen, and identify fungal isolates from the soil and bark of trees from three locations in KwaZulu-Natal, South Africa (29°49ʹ01” S 30°56ʹ41” E; 29°49ʹ03” S 30°56ʹ29” E and 29°16ʹ13” S 31°22ʹ06” E). This paper describes the isolation and screening of thermophilic xylanolytic fungi, as well as the growth parameters for optimal xylanase production by the highest xylanase producer. Selection was made based on the strain’s ability to grow in thermophilic and acidic conditions but directed by the knowledge that if the organisms can grow under these conditions then the enzymes and proteins produced (adaptive evolution) likely function well under these conditions as well. As the xylanase, we were seeking was for an animal feed application requiring enzymes that could function in these conditions our focus remained on the *T. harzianum* strain as it met our requirements even though it is a much studied strain.

## 2. Materials and methods

### 2.1 Isolation, growth, and maintenance of bacteria and fungi

Soil and bark samples were collected from three local sites i.e. the University of KwaZulu-Natal (Westville) close to the Chemistry (29°49ʹ01” S 30°56ʹ41” E) and Microscopy (29°49ʹ03” S 30°56ʹ29” E) disciplines and a town known as Darnall in the north coast region (29°16ʹ13” S 31°22ʹ06” E) in KwaZulu-Natal, South Africa. Three tree species were selected for the tree bark samples; *Acer neguno* (29°49ʹ01” S 30°56ʹ41” E), *Juglans regia* (29°49ʹ03” S 30°56ʹ29” E) and *Citrus limon* (29°16ʹ13” S 31°22ʹ06” E). Soil microbial properties vary widely, both spatially and temporally. Therefore, soil still remains an attractive source of microorganisms with desirable properties. It is important to carefully collect soil samples for bioprospecting microbial enzyme producers according to a given objective or hypothesis. However, in the interest of obtaining many different types of organisms isolation was conducted from soil and tree bark samples that were obtained from different sites as a simple random sampling technique (Lorenz and Dick 2011). Using sterile beakers and spatulas, 1 g of soil and bark samples were transferred to 10 ml sterile tubes. Ten-fold serial dilutions were performed, thereafter 0.1 ml aliquots from each dilution were spread plated on potato dextrose agar (PDA) and incubated at 30°C for 5 to 7 d. Pure isolates were obtained from each dilution plate (Mohammed 2013). Short term (working) stocks were prepared with fungal isolates at 4°C that were previously inoculated and grown for 5 day at 30°C. For the medium term stocks, PDA slants were prepared and left to solidify at room temperature in 15 ml falcon tubes. Fungal cultures were streaked onto the PDA slants, grown for 5 days at 30°C followed by addition of sterile mineral oil to cover the fungal mycelium and storage at 4°C. Long term stocks were prepared by washing fungal spores from the 5-day PDA plates with distilled water and adding 50% glycerol in a 1:1 ratio to the spore suspension and storing at −20°C and −80°C.

### 2.2 Screening for enzyme activity

The screening strategy used was based on a combination of approaches reported in literature (Gautam et al. 2015; da Silva Menezes et al. 2017).

#### 2.2.1 Primary screening of isolates

Qualitative screening was conducted by first growing the fungi on substrate agar plates containing (g/L): 0.5 g NaCl, 1 g KH_2_PO_4_, 0.5 g MgSO_4_, 0.01 g MnSO_4_, 0.3 g NH_4_NO_3_, 0.01 g FeSO_4_, 6 g bacteriological agar supplemented with 1% (w/v) beechwood xylan. Plates were inoculated in the centre with the microorganism and incubated at 30°C for 5 to 7 days, then stained with 0.1% Congo red for 10 min and destained with 1 M NaCl for 15 min to observe and measure halo diameters for xylanase activity (Mosina et al. 2017).

#### 2.2.2 Secondary screening of isolates

The selected xylanase-producing isolates (after primary screening) were inoculated into potato dextrose broth and incubated at 30°C for 7 days at 200 rpm in a shaking incubator (New Brunswick Scientific, incubator shaker series, Innova 44). The cultured media were then centrifuged (Eppendorf Centrifuge 5418, Germany) at 1,6873 × *g* for 10 min. Using sterile pipette tips, 5 mm wells were made on substrate agar plates as prepared above. The supernatants containing the crude enzymes were dispensed into the wells and the plates incubated at 30°C for 2 to 3 days, after which they were stained, destained, and analysed as described previously in 2.2.1.

#### 2.2.3 Tertiary (quantitative) screening of isolates

The isolates that showed high xylanase activity were subjected to quantitative screening after cultivation in a nutrient salt solution (NSS) medium [(g/L): 0.005 g CaCl, 0.23 g KH_2_PO_4_, 0.05 g MgSO_4_, 0.005 g NaNO_3_, 0.002 g ZnSO_4_, 0.009 g FeSO_4_, 0.23 g KCl, 7 g peptone, and 20 g wheat bran]. Erlenmeyer flasks (250 ml) containing 50 ml of the medium were each inoculated with two 5 mm fungal plugs from a 5-day-old plate culture and incubated at 30°C at 200 rpm for 7 d in a shaking incubator (New Brunswick Scientific, incubator shaker series, Innova 44, Germany). Cultured media were removed after the incubation period and the cell-free supernatant was recovered by centrifuging samples at 16,873 *× g* for 10 min (Eppendorf Centrifuge 5418, Germany). Xylanase activity was determined as described below 2.2.4.

#### 2.2.4 Xylanase assay

Xylanase activity was quantified using the DNS assay for reducing sugars according to the method of Miller (1959). The reaction included 600 µl of 1% (w/v) beechwood xylan (1 g in 100 ml of citrate buffer, pH 5) which was placed in 15 ml test tubes and 66.67 µl of the enzyme was added. The reaction mixture was incubated in a water bath at 55°C for 15 min and terminated by adding 1 ml 3,5-dintrosalicylic acid (DNS) reagent to the reaction mixture and then heated for 5 min at 100°C in a water bath. The absorbance was read at 540 nm using a spectrophotometer (Shimadzu UV-1800, Japan) to determine the concentration of sugar released by the enzyme. One unit (U) of xylanase was defined as the amount of enzyme that released 1 µmol xylose as reducing sugar equivalents per min under the specified assay conditions.

### 2.3 Identification of the unknown isolates

The identification of the unknown isolates was accomplished by: (2.3.1) isolation of the genomic DNA; and (2.3.2) amplification of the 18S rRNA ITS2 region; sequencing and BLAST analysis of the 18S rRNA gene.

#### 2.3.1 Genomic DNA extraction

Genomic DNA isolation was performed using the ZR Soil Microbe DNA Miniprep^TM^ kit (Zymo Research, USA) according to the instructions provided by the manufacturer. After extraction, gDNA samples were stored at −20°C. A gDNA concentration of 58.6 (CB1), 13.1 (CB2), 33.9 (PB7), 8.1 (PS3) and 44.7 µg/ml (MS5) was indicative of successful DNA extractions and was used as template DNA in PCR reactions.

#### 2.3.2 Polymerase chain reaction amplification of the 18S ribosomal RNA ITS2 region and identification

**Figure 1:**
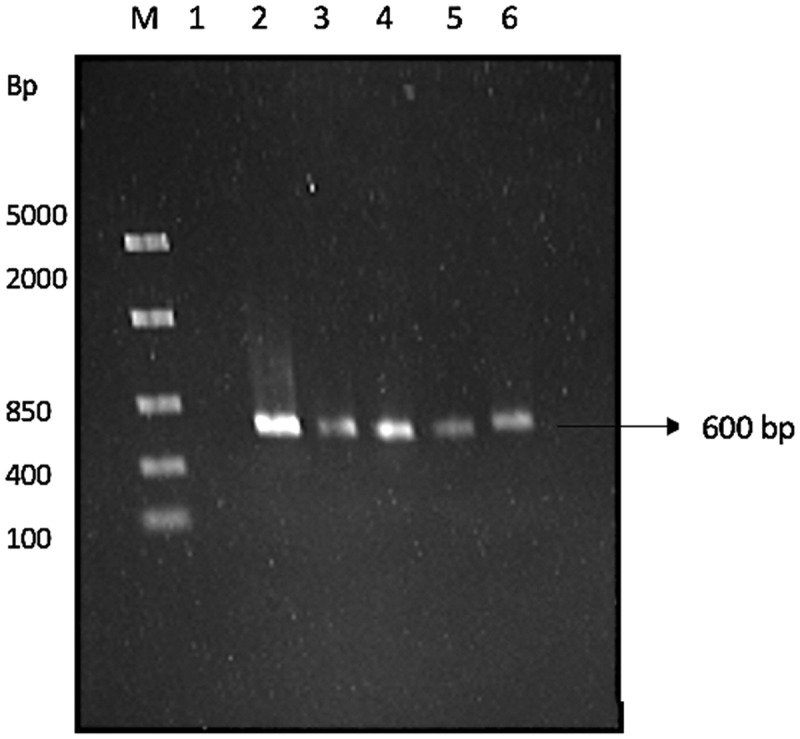
Agarose (1%) gel showing 18s rRNA amplicon. lane M: marker, FastRuler middle range molecular weight ladder (Thermo Sientific, USA), lane 1: negative control, lane 2: CB1, lane 3: CB2, lane 4: PB7, lane 5: PS3, and lane 6: MS5, 600 bp between ITS5 and ITS4 region.

Universal fungal primers for the ITS2 region of the 18S rRNA were used for the PCR reaction. The ITS primer pair used were: forward: ITS5F (5’-GGAAGTAAAAGTCGTAACAAGG–3’) and reverse: ITS4R (5ʹCTCCTCCGCTTATTGATATGC−3’) (White et al. 1990). The PCR reaction consisted of template DNA, 2.5 μM forward and reverse primers, 25 mM MgCl_2_, 10 mM dNTPs, Taq DNA polymerase, 10 mM buffer (Thermo Scientific, USA). The reaction mixture was brought up to a volume of 50 μl using nuclease-free water. Amplification was conducted in a T100 Thermal Cycler (Bio-Rad, USA) under the following thermal cyclic conditions: initial denaturation at 95°C for 2 min followed by 25 cycles of denaturation at 95°C for 30 s, annealing at 53°C for 45 s, and extension at 72°C for 1 min. Thereafter, a final extension step was performed at 72°C for 8 min. The PCR products were then subjected to electrophoresis on a 1% agarose gel, which was run at 90 V for 45 min and then stained with ethidium bromide (0.5 μg/ml). The presence of the PCR amplicon was confirmed and sent to Inqaba BioTec for sequencing. The sequences was cleaned using DNA Man, and thereafter the consensus sequence was used to identify the isolates by BLAST analysis, using the NCBI database (Tuohy et al. 1993). The ITS2 region of the 18S ribosomal RNA was amplified as shown in Figure 1, in which an expected 600 bp amplicon was obtained.

### 2.4 Effect of pH and incubation temperature on xylanase production by MS5

The effect of pH and incubation temperature on xylanase production was studied according to Bhavsar et al. (2016) to aid in the selection of the most promising isolate. High temperatures could result in evaporation and drying of the wheat bran. This was avoided by using a larger total volume of medium and an equivalent inoculum size. The effect of pH on xylanase production was determined in NSS media prepared in buffers ranging from pH 4.0 to 10.0 by adjusting the pH using 1 M HCl and 1 M NaOH. Erlenmeyer flasks (250 ml) containing 50 ml of the medium were each inoculated with two 5 mm fungal plugs from 5-day-old plate cultures and incubated at 30°C at 200 rpm for 7 days in a shaking incubator (New Brunswick Scientific, incubator shaker series, Innova 44, Germany). The pH was not maintained however to verify that the pH was the same as the initial, the pH was measured towards the end of fermentation and compared to the initial pH (4.0–10.0).

The effect of temperature on xylanase production was studied by inoculating a 5-day-old culture into the NSS medium prepared with the pH buffer that resulted in the best enzyme activity determined previously and incubated at 20°C to 80°C for 7 days at 200 rpm. Xylanase assays were performed as described previously and results were reported. The catalytic temperature during the assays in was controlled by a CPS Controller (Shimadzu CPS-240A, Kyoto Japan) attached to the spectrophotometer. The isolate that resulted in the highest enzyme activity was selected for further studies.

### 2.5 Phylogenetic analysis and morphological studies of MS5

#### 2.5.1 Phylogenetic analyses

**Figure 2:**
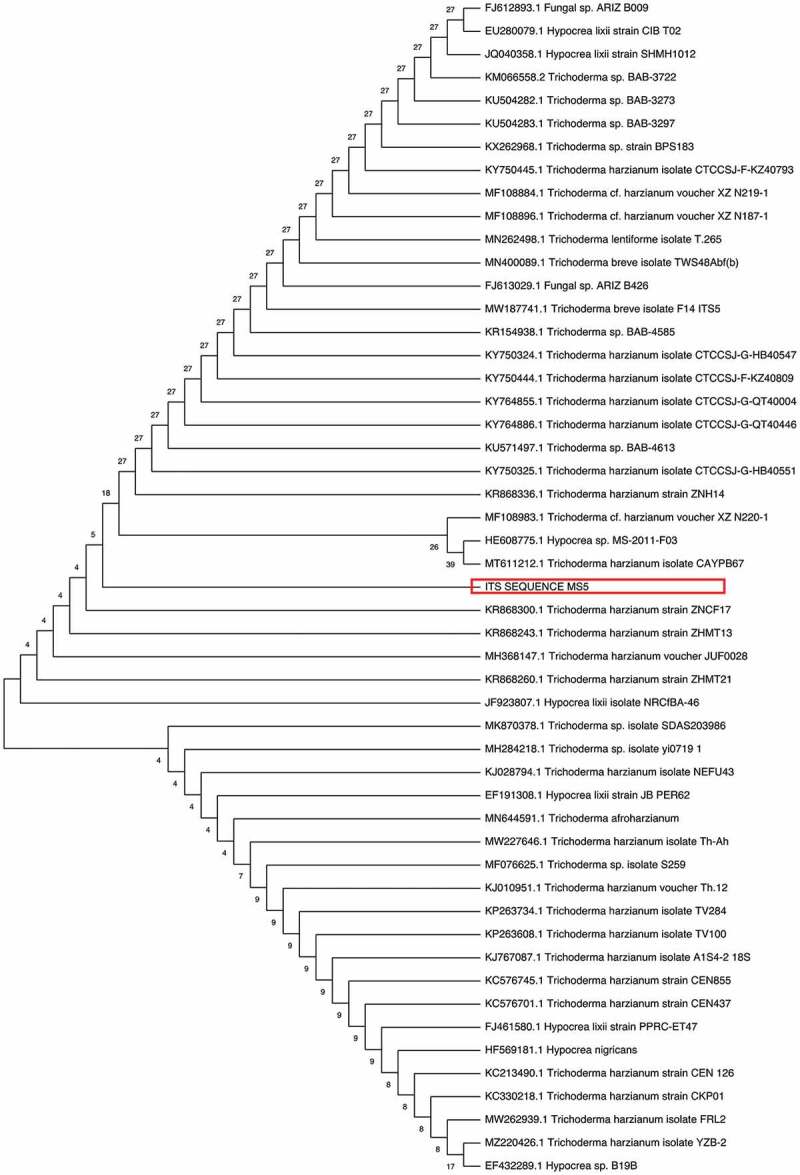
Phylogenetic analysis of the 50 isolates based on alignment of the nucleotide sequences of xylanases including the its sequence of selected MS5 isolate with Mega11. The microbial species, strain name and accession number are presented. Numbers on branches indicate bootstrap support.

The 18S rRNA sequence of the isolate was compared with other closely related strains using BLAST and NCBI GenBank database. Alignment and the phylogenetic tree were constructed using MEGA 11 software. The neighbour-joining (NJ) tree of the isolate was evaluated using 100 bootstrap replications (Tamura et al. 2011). The phylogenetic tree included 51 nucleotide sequences obtained from NCBI Blast (2.5.2). The phylogenetic tree was constructed using the neighbour-joining method (Telles et al. 2018) (Figure 2).

#### 2.5.2 Morphological characterisation using light microscopy

The morphological characteristics of the *T. harzianum* strain were examined using a 5-day-old fungal culture. A lactophenol cotton blue wet mount slide was prepared using the method of Leck 1999. The slide was examined using a light microscope (Primostar 415,500–0057-000, Germany) to examine the structures. The photomicrographs were recorded with an iPhone 6s camera.

### 2.6 Time course for optimal enzyme production

To determine the time required for optimal enzyme production, 50 ml NSS (pH 5.0) was dispensed into 250 ml Erlenmeyer flasks, inoculated with two 5 mm plugs of a 5-day-old fungal culture, and incubated at 200 rpm at 70°C in a shaking incubator (New Brunswick Scientific, incubator shaker series, Innova 44, Germany). Samples were collected every 24 h and centrifuged at 1,6873 ×* g* for 10 min and xylanase activity quantified. All experiments were carried out in duplicate and analytical assays on samples in duplicate yielding quadruplicate results (Cunha et al. 2018).

### 2.7 Effect of agitation on xylanase production

The effect of agitation conditions on xylanase production during submerged shake flask fermentation was studied in Erlenmeyer flasks (250 ml) containing 50 ml of the medium (pH 5.0) inoculated with two 5 mm fungal plugs from a 5 d old plate culture and incubated at 70°C in a shaking incubator (New Brunswick Scientific, incubator shaker series, Innova 44, Germany) at different rpm (120, 140, 160, 180 and 200). All experiments were carried out in duplicate and analytical assays on samples in duplicate yielding quadruplicate results (Cunha et al. 2018).

### 2.8 Effect of different carbon and nitrogen sources on xylanase production

The effect of carbon and nitrogen sources in the NSS was determined. Different carbon sources (1% w/v) such as wheat bran, glycerol (v/v), glucose, sucrose, maltose, and lactose (Okafor et al. 2007; Pandey et al. 2012) and different nitrogen sources (1% w/v) such as peptone, ammonium sulphate, ammonium acetate, casein, glycine, and yeast extract (Gomaa 2013; Ajijolakewu et al. 2017) were used to determine maximal enzyme production under optimised conditions for the other parameters. Once the optimal carbon and nitrogen sources were determined, concentrations between 0.5% and 2% (w/v) were used to determine the optimal concentration for maximum xylanase production.

### 2.9 Biochemical characterisation of crude enzyme

#### 2.9.1 Determination of pH and temperature optima of crude xylanases

To determine the optimum pH values for xylanase activity, different pH buffers were required with 1% substrate for the enzyme reaction at 55°C. The following buffers were used: 0.1 M sodium citrate buffer (pH 3.0–5.0), 0.1 M potassium phosphate buffer (pH 6.0–8.0) and 0.1 M Glycine-NaOH buffer (pH 9.0–10.0) (Franco et al. 2004). All buffers were adjusted to the required pH by using 0.1 M HCl and 0.1 M NaOH as the acid and base. Thereafter, the reaction was stopped by adding DNS and incubating assays at 100°C (Miller 1959).

The optimum temperature of xylanase activity was determined by incubating the enzyme with the optimum pH buffer (pH 6.0 and 8.0) and substrate (1%) in a temperature range between 40°C and 90°C for the reaction time. Thereafter, the enzyme was assayed using the DNS method (Miller 1959).

### 2.9.2 pH and temperature stability of crude xylanase

The pH stability of the enzyme was determined at both pH optima (pH 6.0 and 8.0) by incubating the enzyme in the respective buffers for 4 h at optimum temperature with aliquots removed every 30 min (Abo-Elmagd 2014). Thereafter, xylanase activity was assayed using the DNS method (Miller 1959) and reported as residual activity (%).

Temperature stability of xylanase was determined by preincubating the enzyme at the different temperature optima (55°C, 60°C, 70°C and 75°C) with aliquots collected every 30 min for 4 h for determination of residual activity that was reported as a mean (n = 3) ± SD.

#### 2.9.3 SDS-PAGE

SDS-PAGE was carried out according to Laemmli (1970). A 12% denaturing polyacrylamide gel, which contained SDS was prepared. Following electrophoresis at 50 V for 4 h, the gel was stained with Coomassie Brilliant Blue for 15 min and destained overnight in a de-staining solution. In order to visualise the proteins and determine the molecular mass of the proteins using standard molecular weight markers. To allow for the protein to be more visible, silver staining was performed according to the manual instructions (SilverQuest^TM^ Silver Staining kit, Thermo Fisher Scientific, LC6070).

#### 2.9.4 Substrate native PAGE

Native-PAGE was conducted at room temperature using a 15% polyacrylamide gel supplemented with 1% (w/v) beechwood xylan. Following electrophoresis at 50 V for 4 h, the xylan gel was incubated at optimum temperature (70°C) in pH 5.0 citrate buffer for 1 h. The gel was stained with 0.1% (w/v) Congo red for 15 min at room temperature and destained with 1 M NaCl for 10 min. The protein bands associated with xylanase activity were visualised as clearing zones against a background (Goluguri et al. 2016).

### 2.10. Statistical analysis

Data presented in this paper show the mean of four replicates with their standard deviation (mean ± SD). Results were analysed statistically by Microsoft Excel.

## 3. Results

### 3.1 Screening for enzyme activity

**Figure 3:**
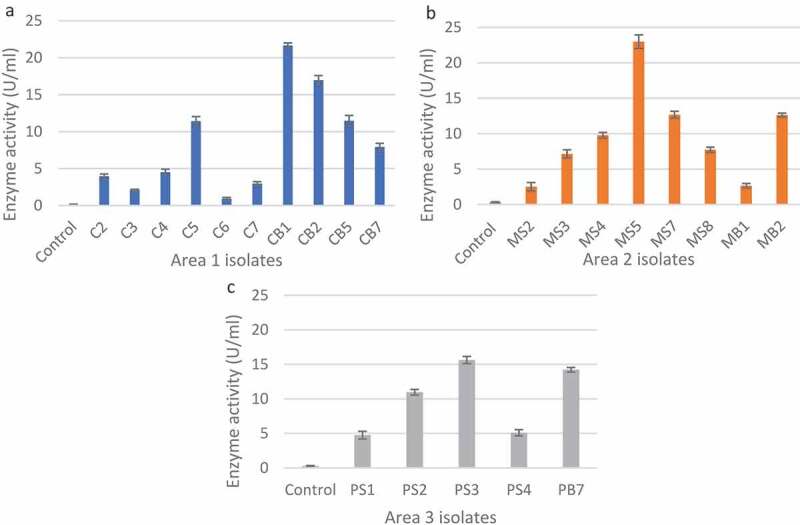
Xylanase activity of the fungal isolates from the different sample areas based on the 3,5-dintrosalicylic acid assay for reducing sugars. a: Area 1, b: Area 2, and c: Area 3.

A total of 48 isolates were obtained from the three sites, with similar isolates obtained from soil and bark samples within the three sites. From all the isolates, 33 were selected for secondary screening based on the size of the clear zones on xylan agar plates (Supplementary Tables 1–3). The unhydrolysed, Congo red-stained xylan medium appeared dark red as seen in the negative control (Table 1). The 23 isolates (Supplementary Tables 4–6) with the largest zones of clearance (Figure 3) were selected for quantitative analysis. In Figure 3, isolates CB1 (21.67 U/ml), CB2 (16.98 U/ml), MS5 (22.98 U/ml), PS3 (15.64 U/ml) and PB7 (14.22 U/ml) produced the highest enzyme activity compared to the others. Fungal morphology on PDA and xylanase activity on substrate agar plates of the five isolates with the highest enzyme activity from Figure 3 are summarised in Table 1.

**Table 1.** Fungal isolates substrate agar screening results for xylanase activity.

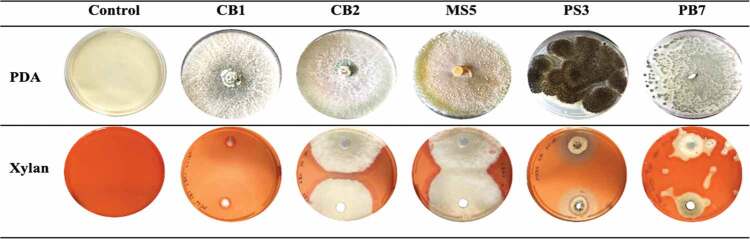

### 3.2 Effect of pH and incubation temperature on xylanase production

**Figure 4:**
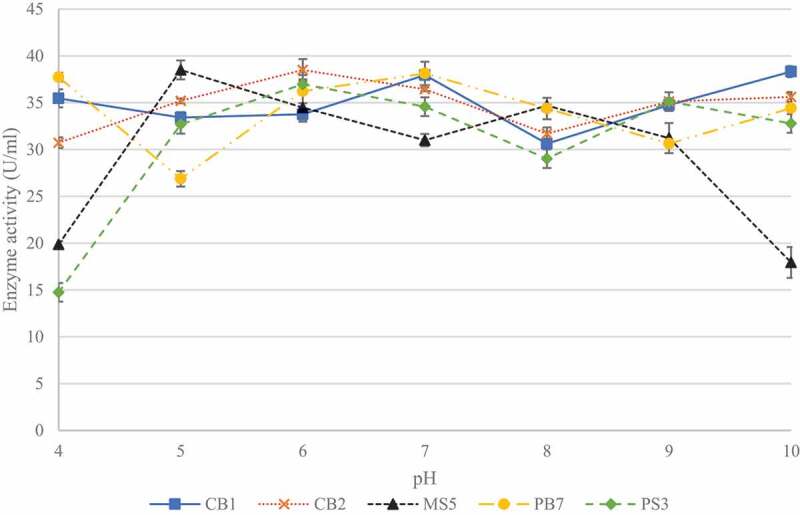
Effect of pH on xylanase production for the five selected fungal isolates, produced during submerged fermentation at 30°C and 200 rpm. Data points represent the means ± SD (n = 4).

Five of the 23 isolates, with the highest activities (CB1, CB2, MS5, PS3, and PB7) were selected for pH and temperature studies in order to determine which isolate would be the best to select for future studies. To determine best conditions for enzyme production, the effect of pH was determined between pH 4.0 and 10.0 (Figure 4). Isolate CB1 produced the highest enzyme activity (38.32 ± 0.89 U/ml) when grown in the medium at pH 10, with elevated enzyme activity at pH 7.0 and 9.0. Isolates CB2 and MS5 produced the highest enzyme activities of 38.49 ± 1.16 U/ml and 38.50 ± 0.76 U/ml at pH 6.0 and 5.0, respectively. Isolate PB7, produced highest enzyme activity (38.12 ± 0.79 U/ml) at neutral pH. Lastly, for isolate PB7, the highest enzyme activity (36.96 ± 0.32 U/ml) was observed at pH 6.0.

**Figure 5:**
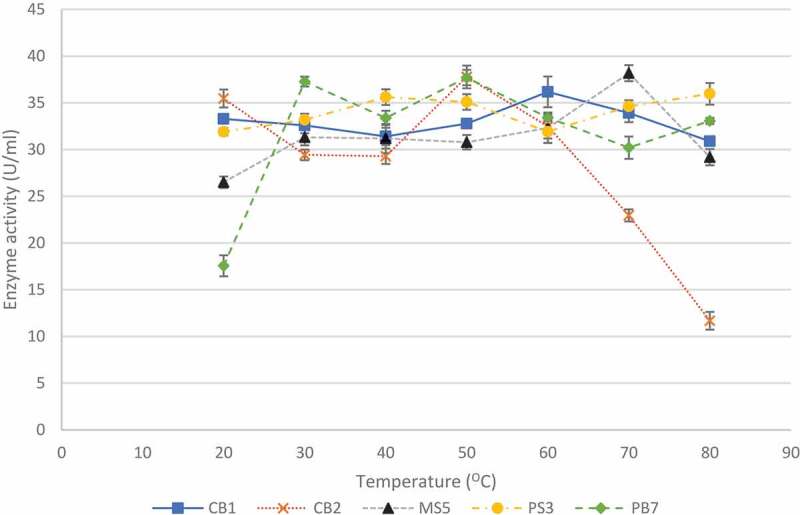
Effect of temperature on xylanase production by the five selected fungal isolates, during submerged fermentation at their optimum pH and 200 rpm. Data points represent the means ± SD (n = 4).

The effect of temperature on enzyme production is shown in Figure 5. Temperatures between 20°C and 80°C were tested as this was the range tested in previous studies (Yadav et al. 2018). For isolate CB1, enzyme activity was highest (36.17 ± 1.65 U/ml) at 60°C and for isolates, CB2 and PB7, the highest activities of 37.77 ± 0.85 U/ml and 37.69 ± 0.82 U/ml, respectively, were observed at 50°C. Isolate MS5 produced the highest enzyme activity (38.17 ± 0.86 U/ml) at 70°C, and isolate PS3 appeared to be an extreme thermophile and produced the highest (35.96 ± 1.16 U/ml) enzyme activity at 80°C with similar activities at 40°C and 50°C (35.61 ± 0.84 U/ml and 35.01 ± 0.84 U/ml). Isolate MS5 was selected for further studies due to its thermophilic properties and acidic conditions.

### 3.3 Identification of five fungal isolates and morphological studies of selected isolate

#### 3.3.1 Identification of fungal isolates

The PCR amplicon was sent to Inqaba BioTec for sequencing and the consensus sequences were submitted to National Centre for Biotechnology Information (NCBI) database to determine the identity of the unknown isolates. Isolate CB1 had a 99.83% identity with *Hyprocrea lixxi* strain TU Graz 3TSM1 and CB2 a 100% identity to *Trichoderma atroviride* strain CUZFVG243. Isolates PB7 and PS3 are both *Aspergillus* sp. with a 99.49% and 99.83% identity to *Aspergillus fumigatus* CK392 and *Aspergillus welwitschiae* SFC102281, respectively. Although sequencing and BLAST analysis resulted in a low (45%) percentage coverage the ID of the unknown isolate MS5 was revealed to be *Trichoderma harzianum* strain with a 99.83% identity to the *Trichoderma harzianum* ZNH14 strain (Table 2).

**Table 2.** Identification of unknown isolates.

Isolate	Identity	Max score	Total score	% Coverage	E.value	% Identity	Accession number
CB1	*Hypocrea lixii* TU Graz 3TSM1	1096	2096	94	0	99.83	EU871017.1
CB2	*Trichoderma atroviride* CUZFVG243	1075	2107	96	0	100	KC884783.1
PB7	*Aspergillus fumigatus* CK392	1066	2111	98	0	99.49	MK439477.1
PS3	*Aspergillus welwitschiae* SFC102281	1068	2046	94	0	99.83	MH374611.1
MS5	*Trichoderma harzianum* ZNH14	1110	2129	45	0	99.83	KR868336.1

#### 3.3.2 Phylogenetic analysis of isolate MS5

Phylogenetic analysis plays an important role in understanding the current research in biological processes (the evolution of species, populations and genes) and became an important data source for how traits evolve over time, the order in which interrelated traits evolve and the influence of an ecology on the evolution of traits. The evolutionary relationship between species is generally reflected in the form of phylogenetic trees (Chen and Zhuang 2017). There were 46 branches originating from the original root. All the *T. harzianum* isolates do not form a single taxon but form sister taxa with other *Trichoderma* species. The isolate reported in the current study forms sister taxa with 2 other *T. harzianum* strains as well as a third branch that splits into 2 sister taxa, one being a *Trichoderma* sp. and the other *T. lixii*.

#### 3.3.3 Morphological studies of MS5

**Figure 6:**
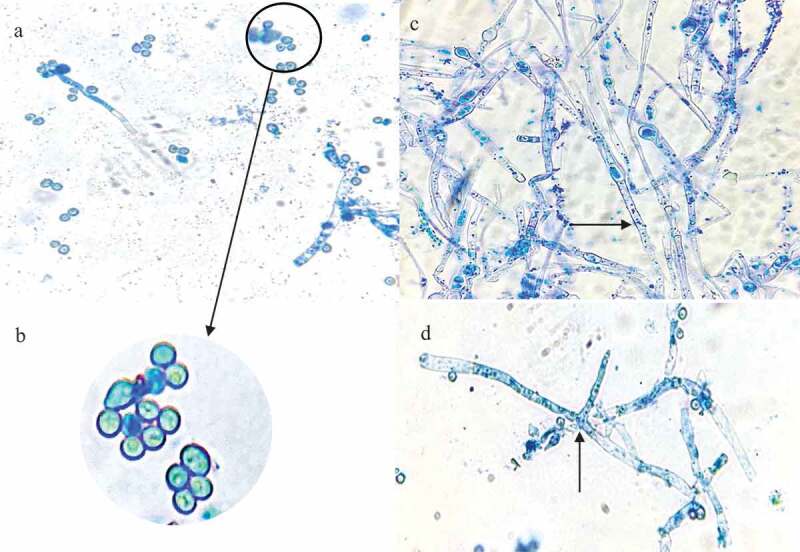
Photomicrographs of a lactophenol cotton blue strain preparation of the identified *Trichoderma harzianum* strain at 1000 x magnification. the black arrows represent, (a) conidia (b) an enlarged image of the conidiospores (c) hyphae, and (d) phialides.

Microscopic examination of the 5-day-old culture using light microscopy showed septate hyphae, conidiospores and phialides (Figure 6). The morphological analysis indicates that isolate MS5 belongs to the *Trichoderma* genus.

### 3.4 Time course studies for optimal xylanase production by *T.* *harzianum*

**Figure 7:**
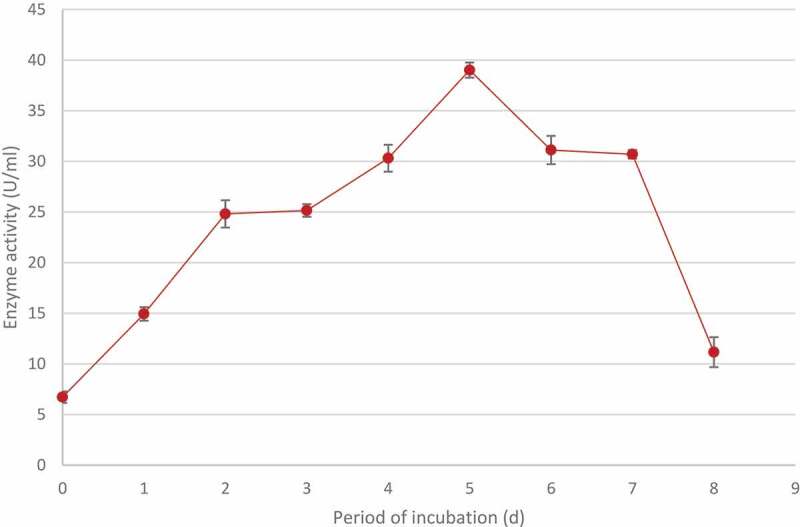
Time course studies showing the optimal period of incubation for maximum xylanase production by identified *Trichoderma harzianum* isolate during submerged fermentation at 70°C, pH 5.0 and standard agitation (200 rpm). Data points represent the means ± SD (n = 4).

The time required for maximal xylanase production was determined by growing the isolateat 70°C at 200 rpm for 1 to 8 days. The highest activity (39.01 ± 0.75 U/ml) was obtained after 5 days. A further increase in the incubation period resulted in a decrease in the enzyme production (Figure 7).

### 3.5 Effect of agitation on xylanase production by *T.* *harzianum*

**Figure 8:**
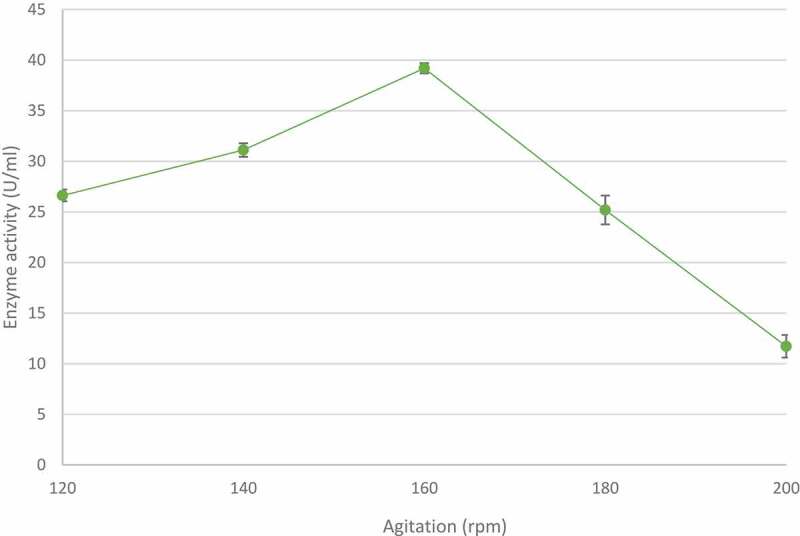
The effect of agitation on xylanase production by the identified *Trichoderma harzianum* isolate, produced during submerged fermentation at 70°C, pH 5.0 for 5 days. Data points represent the means ± SD (n = 4).

The effect of agitation speeds on xylanase production was studied between 120 and 200 rpm at 70°C for 5 days. At the lower agitation rates tested, enzymeproduction was directly proportional to agitation speed with the highest xylanase activity (39.19 ± 0.83 U/ml) observed at 160 rpm (Figure 8). However, a further increase in agitation speed resulted in a sharp and steady decrease in enzyme activity.

### 3.6 Effect of different carbon and nitrogen sources on xylanase production by *T.* *harzianum*

**Figure 9:**
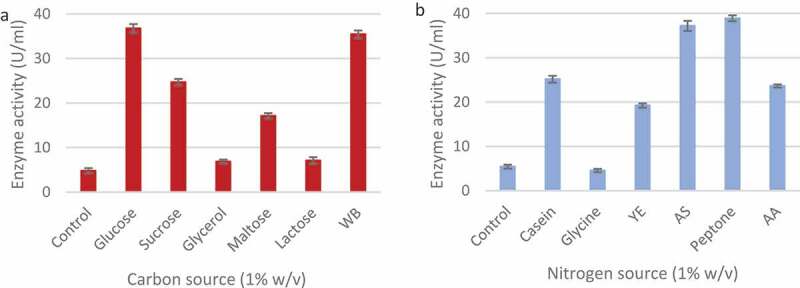
Effect of various carbon (a) and nitrogen (b) sources on xylanase production by identified *Trichoderma harzianum* isolate during submerged fermentation at 70°C, pH 5.0, for 5 days at 160 rpm. WB: wheat bran, YE: yeast extract, AS: ammonium sulphate, and AA: ammonium acetate. columns represent the means ± SD (n = 4)

The effect of carbon (Figure 9a) and nitrogen sources (Figure 9b) in the growth medium were studied to order to maximise enzyme production. It was clear that the *T. harzianum* strain produced the highest xylanase levels when supplemented with glucose (36.76 ± 0.97 U/ml) compared to the control, followed by wheat bran (35.44 ± 0.86 U/ml) and sucrose (24.77 ± 0.71 U/ml). Glycerol had the smallest effect on enzyme production (6.86 ± 0.44 U/ml). Wheat bran was selected as the carbon source for further studies.

**Figure 10:**
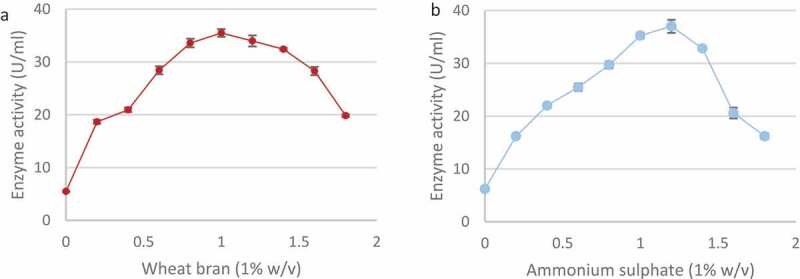
Effect of different wheat bran (a) and ammonium sulphate (b) concentrations (w/v) on xylanase production by the *Trichoderma harzianum* isolate during submerged fermentation at 70°C, pH 5.0, for 5 days at 160 rpm. Data points represent the means ± SD (n = 4).

Of the nitrogen sources tested, peptone produced the highest enzyme activity (38.9 ± 0.67 U/ml) followed by ammonium sulphate (37.20 ± 1.15 U/ml) and casein (25.16 ± 0.77 U/ml) (Figure 9). The addition of glycine proved to be inhibitory (4.53 ± 0.38 U/ml) as it resulted in lower enzyme activity compared to the control (5.42 ± 0.44 U/ml). Ammonium sulphate was selected as the nitrogen source for further studies. A range of wheat bran and ammonium sulphate concentrations were tested in order to establish best concentrations for enzyme production. As shown in (Figure 10) the highest xylanase levels were produced in 1% (w/v) wheat bran (35.49 ± 0.72 U/ml) and 1.2% (w/v) ammonium sulphate (37.01 ± 1.24 U/ml).

### 3.7 Biochemical characterisation of crude xylanases

#### 3.7.1 Determination of pH and temperature optima for enzyme activity

**Figure 11:**
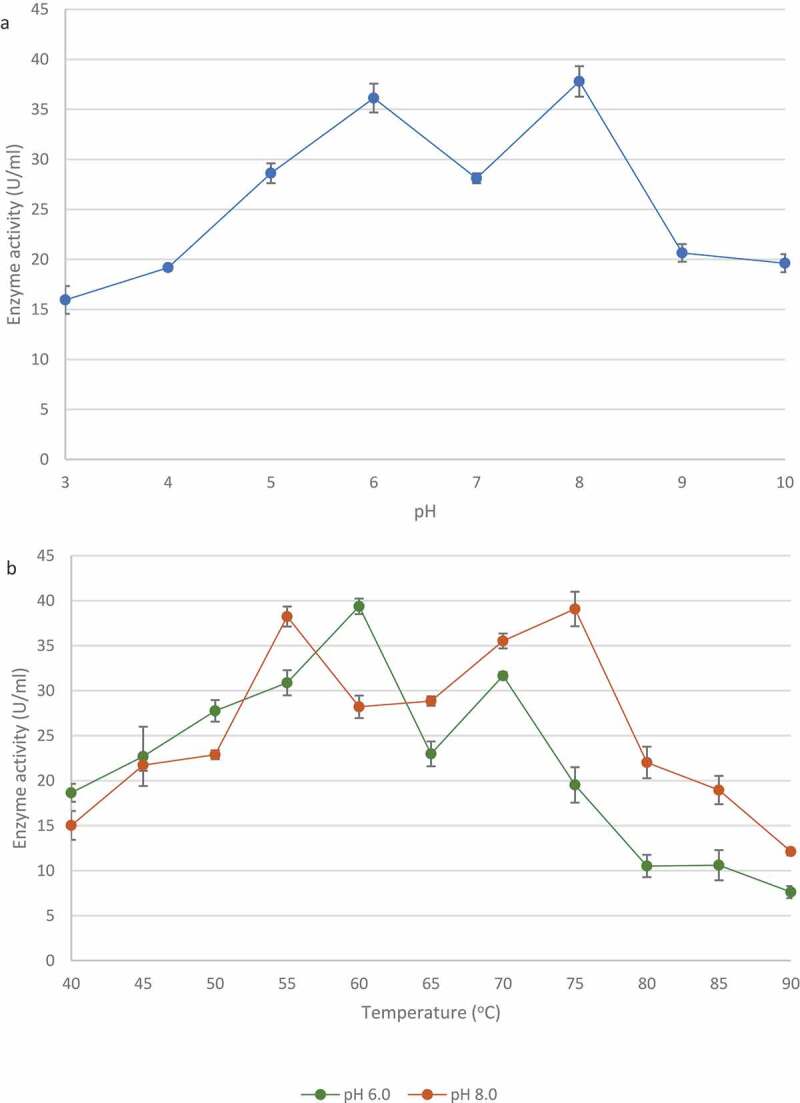
Effect of pH (a) and temperature (b: at pH 6.0 and pH 8.0)) on the activity of xylanase from the identified *Trichoderma harzianum* isolate crude extracellular supernatants. Data points represent the means ± SD (n = 3).

Various pH affected the activity of xylanase from *T. harzianum*. The highest xylanase activity was seen at pH 8.0 (37.80 ± 1.53 U/ml) and a second peak was observed at at pH 6.0 (36.13 ± 1.44 U/ml) (Figure 11a). Enzyme activity assays were performed at various temperatures at both pH optima. Figure 11b shows that the xylanase activity was the highest at 60°C (39.37 ± 0.86 U/ml) witha second peak at 70°C (31.66 ± 0.41 U/ml) at pH 6.0, whereas at pH 8.0, the xylanase activity was the highest at 75°C (39.08 ± 1.92 U/ml) and peaked again at 55°C (38.24 ± 2.11 U/ml).

#### 3.7.2 Determination of pH and temperature f on enzyme stability

**Figure 12:**
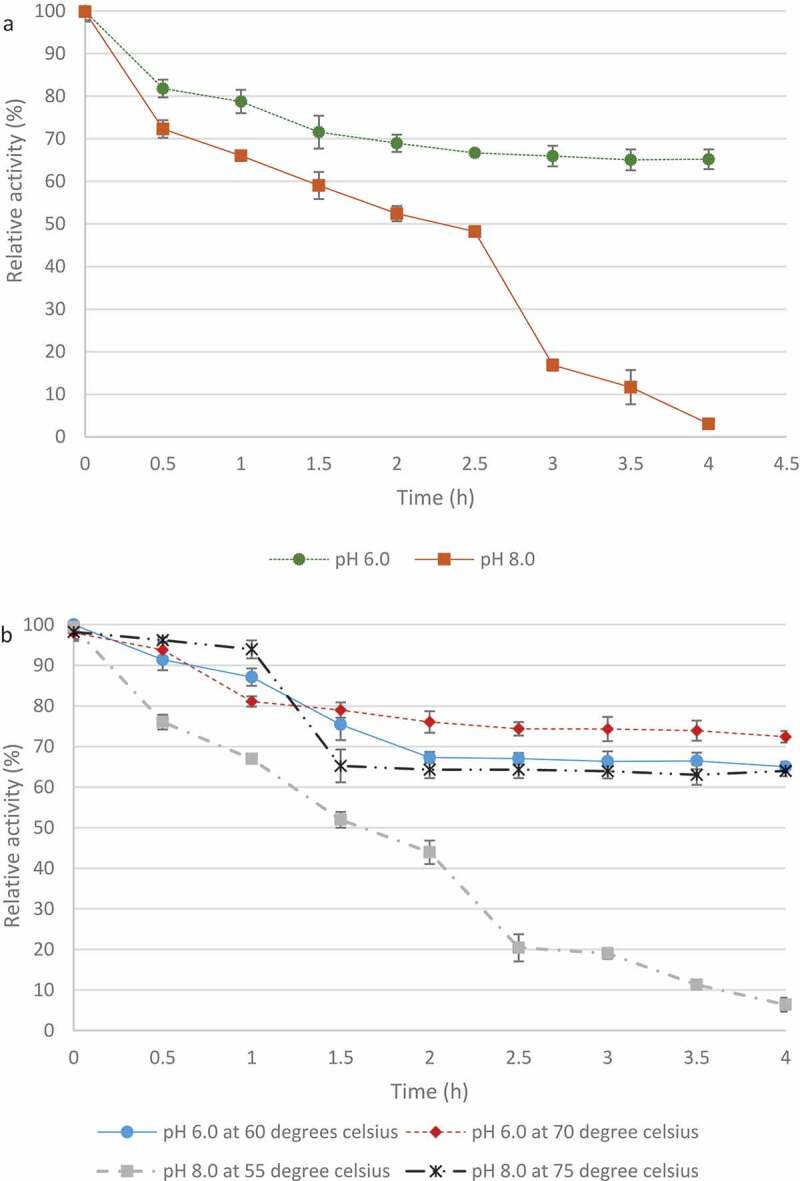
pH (A) and temperature (B) stability of crude xylanases produced by the *Trichoderma harzianum* isolate. Data points represent the means ± SD (n = 3).

The *T. harzianum* xylanase retained >50% maximal activity at pH 6 after 4 h (Figure 12a) whereas at pH 8.0 the enzyme only retained activity for 0.5 h which gradually declined for 2.5 h with sharper decline in activity between 2.5 and 4 h. In Figure 12b, at pH 6.0, the enzyme retained >50% activity after 4 h at both 60°C and 70°C. At pH 8.0, the enzyme retained >50% activity at 75°C for 4 h and lost activity after 4 h at 55°C.

#### 3.7.3 Polyacrylamide Gel Electrophoresis

**Figure 13:**
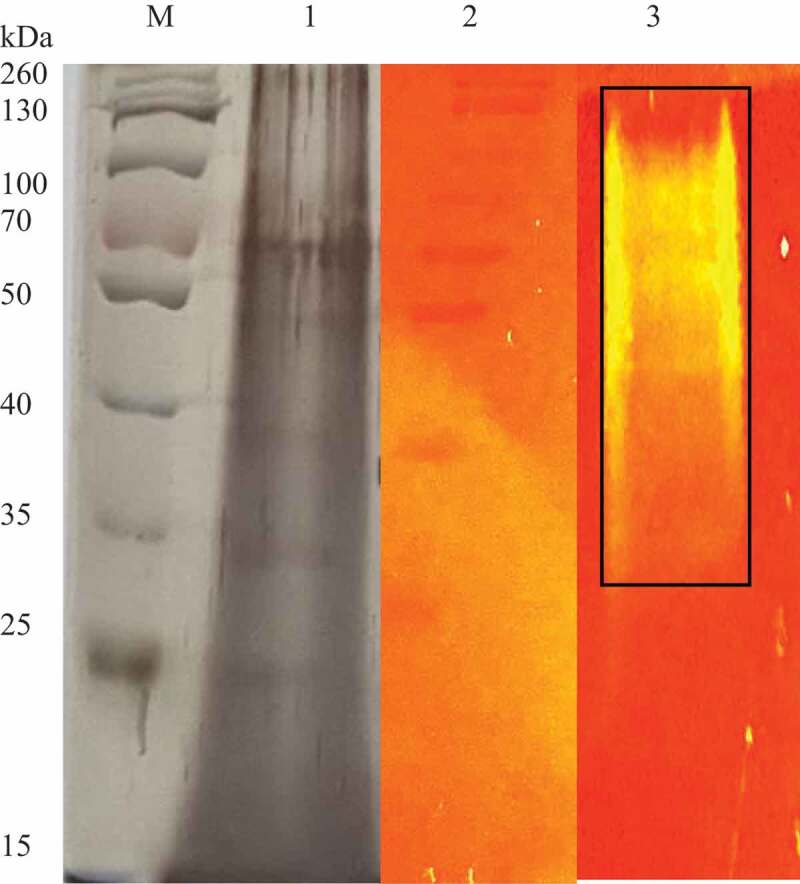
SDS PAGE gel (silver stained) and native substrate PAGE gel (1% xylan) of the *Trichoderma harzianum* crude xylanases. lane M: Spectra multicolour broad range marker (Thermo Scientific, USA), lane 1: crude extract, lane 2: Spectra multicolour broad range marker Thermo Scientific, USA) (stained with Congo red) and lane 3: crude xylanase extract showing zones of clearance.

The SDS PAGE gel of the crude xylanase enzyme (Figure 13) was stained using silver staining, which revealed multiple protein bands in the crude extract. A prominent band with a molecular weight of 70 kDa was observed. The native PAGE gel supplemented with 1% (w/v) beechwood xylan revealed hydrolysis of the substrate as evidenced by the areas with a slightly yellow hue. The majority of the xylanase activity corresponded to the mass of proteins in the top half of the gel between 34 and 130 kDa in molecular weight where brighter bands were observed at a higher molecular weight (55–130 kDa).

## 4. Discussion

In spite of advanced knowledge of microbial xylanases garnered over the past decades, bioprospecting for organisms with xylanases with potentially ideal characteristics for industrial application is ongoing and to this end several factors (location, desired qualities, characteristics of microorganism, etc.) are still significant for the choice of such an organism. In the present study, 46 filamentous fungal isolates were obtained from soil and bark samples and screened for their xylanase activity under submerged fermentation. During the isolation, the growth of bacteria was not intentionally prevented, instead sub culturing was performed from the original plates to obtain pure fungal cultures. Primary screening using qualitative methods is a powerful tool that allows rapid and easy screening of microorganisms for enzyme production. This qualitative substrate agar test indicates positive or negative enzyme production and is invaluable when screening a large number of isolates and quantitative analysis is not required (Pointing 1999). The xylanase-producing isolates displayed clearing zones from a dark red to a light red colour representing efficient xylanase activity. The low enzyme activity displayed by some isolates may be due to the presence of contaminants or enzyme activities being too low for complete hydrolysis of the substrate for visualisation on the substrate agar (Singh et al. 2006). After screening, five isolates were selected for pH and incubation temperature studies for the fermentation medium.

The pH plays a crucial role in nutrient transport across the membrane and the functioning of the microorganism’s enzyme systems and thus influences the growth rate and the levels of enzyme produced (Gupta and Kar 2009). The temperature of the fermentation medium is a vital factor that has a strong influence on product formation (Pathak et al. 2014). Isolate CB1 identified as *Hypocrea lixii* (is a teleomorph of *T. harzianum*), exhibited the highest activity at pH 10 with several other peaks indicating the organism, is possibly an alkaliphile that also produced isoforms with different pH and temperature optima. Temperatures between 20°C and 80°C were tested and all five isolates produced the highest activities at temperatures between 50°C and 70°C. Again, a second enzyme activity peak observed would indicate the presence of more than one isoform. de Oliveira et al. (2014) also reported the presence of isoforms at 55°C at pH 5.0 and at 44°C at pH 3.6 for xylanases produced by *H. lixii*. Isolate CB2, identified as *Trichoderma atroviride*, appeared to be an acidophile and produced the highest activity at pH 6.0. Grigorevski-Lima et al. (2009) reported similar results with optimal pH studies. Isolates PS3 and PB7 displayed highest activity at neutral pH and at pH 6.0, respectively, indicating that the latter could possibly be an acidophile. Both these isolates are *Aspergillus* spp. and Hombalimath et al. (2021) reported similar studies where optimal activity was obtained at pH 7.0. Isolate MS5 showed optimal results at pH 5.0 with a second peak at pH 8.0 representing isoforms was similar to a report by Amore et al. (2015) who obtained maximal activity at pH 7.0 and a second peak at pH 9.0. Most fungi can grow in a wide pH range of 5.0–10.0 (Singh et al. 2006; Abubakar et al. 2013). Generally, the higher xylanase titres (2701 U/g substrate) in fungal systems have been reported to occur at pH 5.0 (Shah and Datta 2005). However, their reported activity was expressed per gram substrate utilised which was reported for lignocellulosic biomass whereas the current study reports it in the conventional manner (U/ml). In addition, many studies report on the purified enzymes or on recombinant enzymes thus activities are higher (Zhang et al. 2010; Yang-yuan et al. 2015; Deshmukh et al. 2016). Xylanases produced by these isolates may have a potential for applications in different sectors, including food and feed, paper and pulp and textile industries that require enzyme to work at high temperatures. The highest xylanase titres in fungal systems have generally been reported to occur at temperatures that are optimal for the growth of cultures in submerged fermentation (Sanghvi et al. 2010). The majority of fungal isolates produce one to three xylanase isoforms (Polizeli et al. 2005; Gong et al. 2018). Lenartovicz et al. (2002) reported that three xylanase isoforms were produced by *Aspergillus fumigatus*. However, *Thermomyces stellatus* and *Scytalidium thermophilium* are reported to have 10 isoforms while *Pseudocercosporella* and *Myceliophthora themophilum* have 14 and 13 isoforms, respectively (Chadha et al. 2019). Multiple forms of xylanases differ in stability, catalytic efficiency, absorption, and activity on substrates (Liao et al. 2015). Badhan et al. (2007) reported that 10 different functionally diverse xylanases were resolved electrophoretically using PAGE/IEF from *Myceliophthora* sp. and they also showed characteristically different activity against unsubstituted xylans, arabinoxylans and methyl-glucouroxylan.

Isolate MS5 was selected for further studies due to its thermophilic properties (temperature optimum of 70°C) and acidic pH optimum of 5.0; these properties were deemed favourable for the conditions of its targeted application in the hydrolysis of animal feed (Rigoldi et al. 2018). The advantages of thermophilic enzymes for conducting biotechnological processes at elevated temperatures are; reducing the risk of contamination by mesophilic microorganisms, decreasing the viscosity of the reaction medium, increasing the bioavailability and solubility of organic compounds, increasing the diffusion coefficient of substrates and products resulting in higher reaction rates (Kambourova 2018).

Azimova et al. (2020) reported that the *T. harzianum* strain UzCF-28 produced xylanases, which was also confirmed by Abbas et al. (2012). The Blast analysis returned a low percentage coverage (45%); however, the ITS2 amplicon was of the recommended size, the E value (Table 2) is 0 despite the low percentage coverage, there was a 99.8% similarity to *T. harzianum* ZNH14 (Chamacho et al., 2009). Filamentous fungi that belong to the *Trichoderma* genus are attractive (Marecik et al. 2018) due to their inducible enzyme systems, the considerable quantities of enzymes secreted by these fungi, including cellulase and hemicellulase cocktails. As a result of their wide spectrum of metabolic activities, *Trichoderma* sp. fungi have found numerous practical applications such as enzyme producers, as bio-fungicides (Mulatu et al. 2021), and in the food industry (Harris and Ramalingam 2010).

Sequences were assembled and aligned using the Mega11 software. All species of the related taxa from Blast analysis were included in the phylogenetic tree, all *T. harzianum* isolates were not grouped together and the species were either scattered among the clades or showed separate terminal branches. Chen and Zhuang (2017) discovered several new *Trichoderma* sp. during a screening exercise conducted on several hundred soil samples in China. They reported that *T. harzianum* formed the largest clade among the green-spored groups that formerly contained 41 species.

The abbreviated tree was constructed using 50 different microorganisms, mostly *T. harzianum* strains. Two predominant sister clades emerge from the root. Both consist of strains of *T. harzianum* but with Hypocrea strains forming part of the same sister clades, such as *T. harzianum* strains as well as *T. lixii* NRCfBA-46 in terms of distance (Chen and Zhuang 2017). Chaverri and Samuels (2002) reported cultures derived from ascospores of *Hypocrea lixii* (*T. lixii*) cultures produced the morphological species *T. harzianum*. The *H. lixii* isolates were shown to group with isolates of *T. harzianum* based on phylogenies of four genes, translation elongation factor-1 α, calmodulin, actin and ITS rDNA, and morphological and cultural data (Chaverri et al. 2003). This could explain the genetic link between the two morphs.

When grown on PDA, the *T. harzianum* strain initially produced a fast-growing white downy mycelium (Table 1) which then changed to yellowish-green and later deep green as it matured. The conidiation predominantly effuse which appeared powdery and granular due to dense conidiation producing woolly and floccose compact tufts and rings with green coloured spores fringed by sterile white mycelium (Gams and Bissett 2002). Microscopic examination of the 5-day-old culture using light microscopy showed septate hyphae, conidiospores and phialides (Figure 6). Conidiospores of *T. harzianum* were formed in pairs along the main branches and axis (Figure 6a). The hyphae were thin and branched (Figure 6c). The phialides branching patterns were verticillate, broad, and branching frequently at approximately 90° one branching verticillate had two to three phialides (Figure 6d). Phialides were characteristically elongate lageniform in shapes. At the end of the phialides, conidia were formed with a globose or subglobose shape to obvoid and are smooth-walled, subhyaline to pale green (Figure 6b). These structures are similar to those described in previous studies (Gams and Bissett 2002).

Production of microbial enzymes is dependent upon various cultural and nutritional factors such as fermentation incubation period, agitation, carbon and nitrogen sources and hence these were studied in order to optimise xylanase production by *T. harzianum*. For the optimal period for enzyme production, the fungal isolate was incubated for a period of 8 d at the optimal pH and temperature obtained previously. The highest enzyme activity (39.01 U/ml) was obtained after 5 days. Similar results were reported by Goyal et al. (2008) where optimal xylanase activity (139.82 U/ml) was obtained between 5 and 7 days of incubation for *T. viride*. After 5 days of incubation, the enzyme activity declined, this could be due accumulation of toxic products in the medium that inhibits fungal growth, repression of enzyme expression by metabolic products or by multiple regulatory mechanisms (Shulami et al. 2014).

Agitation is considered an important factor for microbial growth as it controls the transfer of oxygen, heat, and nutrients from the medium to the microorganism and prevents the clumping together of the mycelia (Ibrahim et al. 2015). Highest enzyme activity (39.19 U/ml) was observed at 160 rpm (Figure 8) with higher agitation speeds resulting in lower xylanase activity. These higher agitation levels may have aggravated cell damage which, in turn, could have led to enzyme production falling off (Zhu et al. 2012). Several reports link increased agitation speeds to high shear stress leading to mycelial rupture and destruction of cellular structures, which decreases both mycelial growth and enzyme production (Bhattacharyya et al. 2008; Singhania et al. 2011). At high agitation rates, laminar flows are generated in a flask, which do not allow absorption of oxygen into the medium although rpm is high, which may cause reduced growth and enzyme production. However, below a certain threshold, lower agitation rates result in reduced mixing of the medium and as a consequence lower oxygen supply to the microorganism, lower growth rates and thus enzyme production.

Carbon and nitrogen sources are necessary for the growth and metabolism of microorganisms. The use of affordable C and N sources is important as these can reduce the cost of production significantly. Surprisingly, the best carbon source for xylanase production from *T. harzianum* was glucose. Carbon catabolite repression (CCR) has to be overcome usually for expression of hydrolytic enzymes (Hu et al. 2021). Basal expression levels of xyn1 coding for xylanase are affected by glucose and repression is reported to be mediated by the binding of the catabolite repressor protein Cre1 to the promoter of xyn 1. Mutants with truncated cre1 escape CCR (Mach et al. 1996). AC et al. (2008) studied xylanase regulation in *Aspergillus phoenicis* by growing the organism in media supplemented with 1% glucose, xylan or xylose. In the first few hours, glucose repressed xylanase production but after 72 h the level of glucose in the culture medium had dropped below 0.05 mg/mL, and some xylanolytic activity was already detectable in this carbon-derepressed culture. We believe that the levels of xylanase obtained on our study were done during the de-repressed phase. The next best substrate was wheat bran while glycerol resulted in the lowest xylanase levels. Low activity could be due to the inability of the fungus to metabolise some substrates. Seyis and Aksoz (2005) tested xylanase production by *T. harzianum* 1073 D3 and reported maximum activity on melon peel (26.5 U/mg of protein) Wheat bran was selected as the carbon source as glucose is not cost-effective. Other enzyme production studies have also shown that wheat bran is a good carbon source for maximal xylanase production (Biswas et al. 2019).

The organic nitrogen source, peptone resulted in the maximum yield of xylanase from *T. harzianum* followed by ammonium sulphate (Figure 9) while xylanase production decreased in the presence of glycine. A review of the literature showed that ammonium sulphate (37.2 U/ml) is an appropriate source of nitrogen for *T. harzianum*, so it was selected as peptone (38.9 U/ml) is not cost-effective (Abdullah et al. 2015; Biswas et al. 2019). Glycerol had the greatest catabolic repression as the carbon source due to its minimal enzyme activity (6.86 U/ml). da Silva Delabona et al. (2016) reported that the use of glycerol to bulk up biomass is a good strategy followed by induction for cellulase production. Glycine as the nitrogen source had the greatest catabolic repression as minimal enzyme activity (4.53 U/ml) was observed.

Biochemical characterisation of xylanase revealed an enzyme with promising properties (high enzyme activity at various pH and temperatures) for future studies. The crude xylanase from *T. harzianum* demonstrated two pH optima: at 6.0 and 8.0 meaning that at least two different isoforms are present. The enzyme displayed greater stability at pH 6.0 and retained more than 50% of its activity over 4 h. However, it was not very stable at pH 8.0, as it lost activity after 1.5 h. These results are similar to those reported by Costa et al. (2019) and Sharma et al. (2016) for xylanase from *T. viride* and thermostable *Fusarium* sp. XPF5. Abo-Elmagd (2014) found that the xylanase from *T. harzianum* MH-20 had optimum activity at pH 5.5 and was stable from pH 5.5–6.5. Temperature can influence reaction rate of an enzyme. Thermal stability of the enzyme was observed at higher temperatures than expected with the xylanase retaining >50% of its activity at 60°C, 70°C and 75°C.

Thermostable and neutrally stable xylanases are beneficial for large scale production as the process would be simplified and save cooling time as well as reduce the problems of possible contamination (Liang et al. 2010) thus reducing costs. However, the target enzyme in the current study is produced under mildly acidic conditions, yet the cost impact may be minimised if acid lysed lignocellulose is used as the carbon source. The multiple high activities at various temperature and pH optima observed suggests that this organism produces multiple xylanases. This has been reported previously (Raj et al. 2013) who also reported multiple xylanases and endoglucanases produced by *Simplicillium obclavatum* MTCC 9604 during growth on wheat bran. Each xylanase may have diverse structures, physicochemical properties and rate of activities.

Several bands at approximately at 70 kDa, 49 kDa, 39 kDa, and 25 kDa (Figure 13) were apparent for the crude *T. harzianum* xlanase de Paula Silveira et al. (1999) and Silva et al. (2015) reported xylanase at 14–19 kDa from *T. inhamatum* and *T. harzianum*. Nathan et al. (2017) reported molecular weights between 14 and 66 kDa for xylanases from *T. viride* VKF3. Native substrate PAGE was performed using a native gel supplemented with xylan substrate and stained with Congo red. This approach and zymogram analysis are widely used for confirmation of xylanolytic activity and to identify the fractions that possess the activity during purification. A range of isoforms were detected (55–130 kDa) as the crude enzyme migrated through the Native PAGE gel and hydrolysed the xylan forming bands of hydrolysis. Nathan et al. (2017) reported xylanase activity at 14 kDa and between 43 and 66 kDa.

*T. harzianum* xylanases with various pH and temperature optima and activities have been studied previously, e.g. one with a lower temperature optimum (22°C) (Azzouz et al. 2020). Bhalla et al. (2015) reported maximal xylanase activity from *Geobacillus* sp. strain WSUCF1 at 60°C (pH 6.0). However, the current study is the first report of a *T. harzianum* strain isolated in South Africa with thermophilic isoforms displaying high activities and different pH optima, which are suitable for applications in animal feed improvement and biofuels production. The isoform produced at acidic optimum (pH 5.0) was targeted in this study for future applications in the feed industry.

The growth conditions of the isolate in submerged fermentation has been performed to find the best conditions for highest xylanase production. Future studies using statistical experiment design (Plackett-Burman and Response Surface Methodology), scaled up production in bioreactors, purification and characterisation of the pure enzyme will be conducted.

## Supplementary Material

Supplemental Material
