# Supplementary material for: Isolation, screening, preliminary optimisation and characterisation of thermostable xylanase production under submerged fermentation by fungi in Durban, South Africa
Source: Mycology. 2022 Jun 20;13(4):271–92. doi: 10.1080/21501203.2022.2079745 (PMC9673795; doi:10.1080/21501203.2022.2079745)
Supplement: Supplemental Material [file TMYC_A_2079745_SM1247.docx]

Table 1: Diameter of clearance zones representing xylanase activity for site 1 (29^o^49'01''S 30^o^56'41'' E)

| **Isolate** | **Activity** |
| --- | --- |
| C1 | _ |
| C2 | ++ |
| C3 | ++ |
| C4 | ++ |
| C5 | ++ |
| C6 | ++ |
| C7 | ++ |
| CB1 | ++ |
| CB2 | ++ |
| CB3 | ++ |
| CB4 | + |
| CB5 | ++ |
| CB6 | + |
| CB7 | ++ |
| CB8 | ++ |
| CB9 | + |
| CB10 | - |
| CB11 | - |

Key: none (-), 1-2 mm (+), >2 (++)

Table 2: Diameter of clearance zones representing xylanase activity for site 2 (29^o^49'03''S 30^o^56'29'' E)

| **Isolate** | **Activity** |
| --- | --- |
| MS1 | _ |
| MS2 | ++ |
| MS3 | ++ |
| MS4 | ++ |
| MS5 | ++ |
| MS6 | + |
| MS7 | ++ |
| MS8 | ++ |
| MB1 | ++ |
| MB2 | ++ |
| MB3 | ++ |
| MB4 | + |
| MB5 | ++ |

Key: none (-), 1-2 mm (+), >2 (++)

Table 3: Diameter of clearance zones representing xylanase activity for site 3 (29^o^16'13''S 31^o^22'06'' E)

| **Isolate** | **Activity** |
| --- | --- |
| PS1 | ++ |
| PS2 | ++ |
| PS3 | ++ |
| PS4 | ++ |
| PB1 | + |
| PB2 | ++ |
| PB3 | ++ |
| PB4 | ++ |
| PB5 | ++ |
| PB6 | + |
| PB7 | ++ |
| PB8 | ++ |
| PB9 | + |
| PB10 | + |
| PB11 | ++ |

Key: none (), 1-2 mm (+), >2 (++)

Table 4: Diameter of clearance zones showing xylanase activity for site 1(29^o^49'01''S 30^o^56'41'' E)

| **Isolate** | **Activity** |
| --- | --- |
| C2 | ++ |
| C3 | ++ |
| C4 | ++ |
| C5 | ++ |
| C6 | _ |
| C7 | _ |
| CB1 | ++ |
| CB2 | ++ |
| CB3 | ++ |
| CB5 | ++ |
| CB7 | ++ |
| CB8 | _ |

Key: none (-), 1-2 mm (+), >2 (++)

Table 5: Diameter of clearance zones showing xylanase activity for site 2 (29^o^49'03''S 30^o^56'29'' E)

| **Isolate** | **Activity** |
| --- | --- |
| MS2 | ++ |
| MS3 | ++ |
| MS4 | ++ |
| MS5 | ++ |
| MS7 | ++ |
| MS8 | ++ |
| MB1 | ++ |
| MB2 | ++ |
| MB3 | ++ |
| MB5 | _ |

Key: none (-), 1-2 mm (+), >2 (++)

Table 6: Diameter of clearance zones showing xylanase activity for site 3 (29^o^16'13''S 31^o^22'06'' E)

| **Isolate** | **Activity** |
| --- | --- |
| PS1 | ++ |
| PS2 | ++ |
| PS3 | ++ |
| PS4 | ++ |
| PB2 | ++ |
| PB3 | ++ |
| PB4 | ++ |
| PB5 | _ |
| PB7 | ++ |
| PB8 | ++ |
| PB11 | ++ |

Key: none (-), 1-2 mm (+), >2 (++)
